# Supplementary material for: snPATHO-seq, a versatile FFPE single-nucleus RNA sequencing method to unlock pathology archives
Source: Commun Biol. 2024 Oct 16;7:1340. doi: 10.1038/s42003-024-07043-2 (PMC11484811; doi:10.1038/s42003-024-07043-2)
Supplement: Supplementary file 2 — Supplementary Information [file 42003_2024_7043_MOESM2_ESM.pdf]

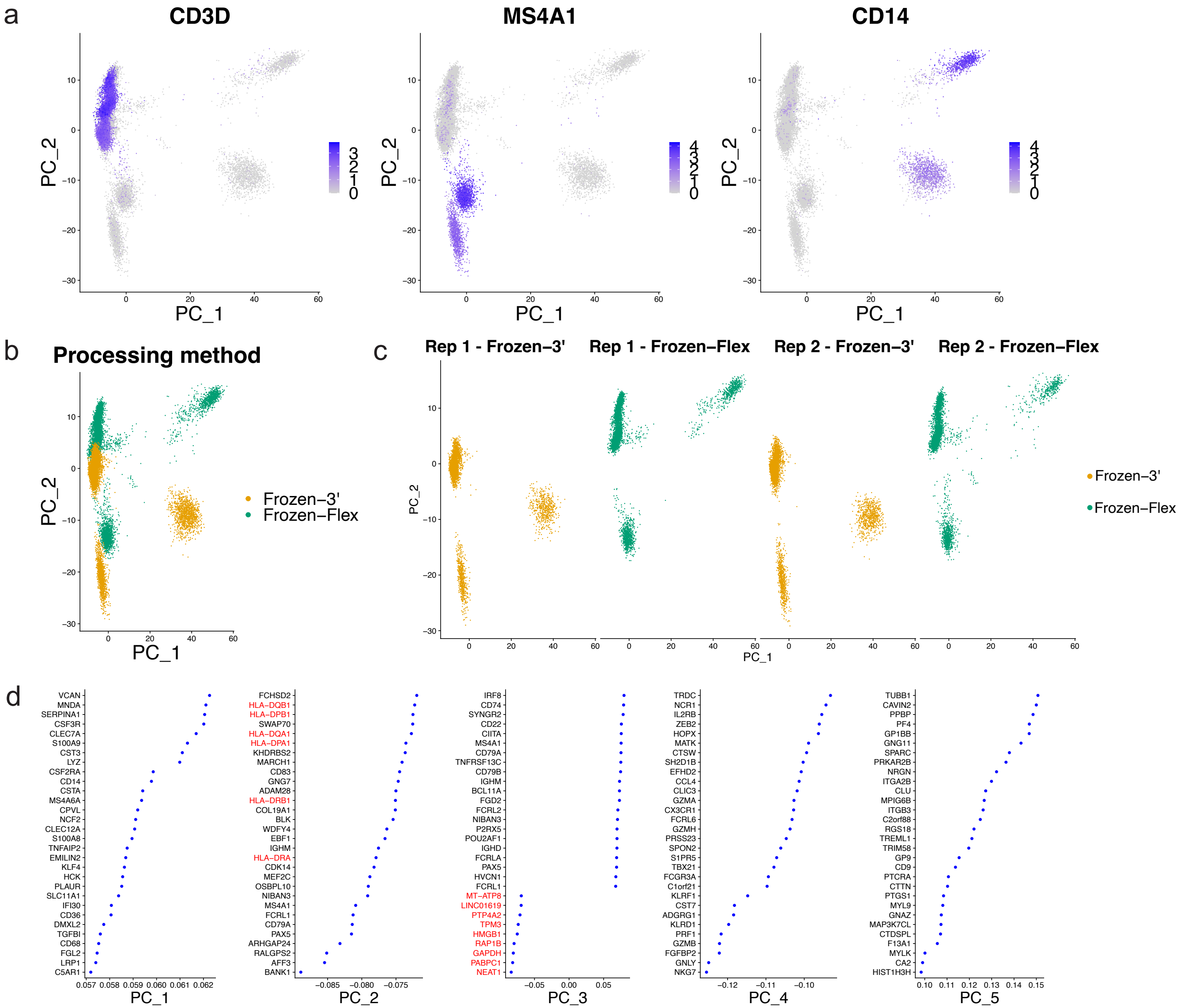

Supplementary Figure 1: 10x Flex and 10x 3' chemistry differ by transcriptomic coverage. (**a**) PCA embedding of unintegrated PBMC data with the expression of selected cell type markers. (**b**) PCA embedding of PBMC data colored by processing methods. (**c**) PCA embedding of unintegrated PBMC data split by sample IDs and processing methods. (**d**) Dotplot showing the contribution of the top 30 genes with the highest absolute contribution to PC1-PC5. Genes absent in the Flex probe panel were colored in red.

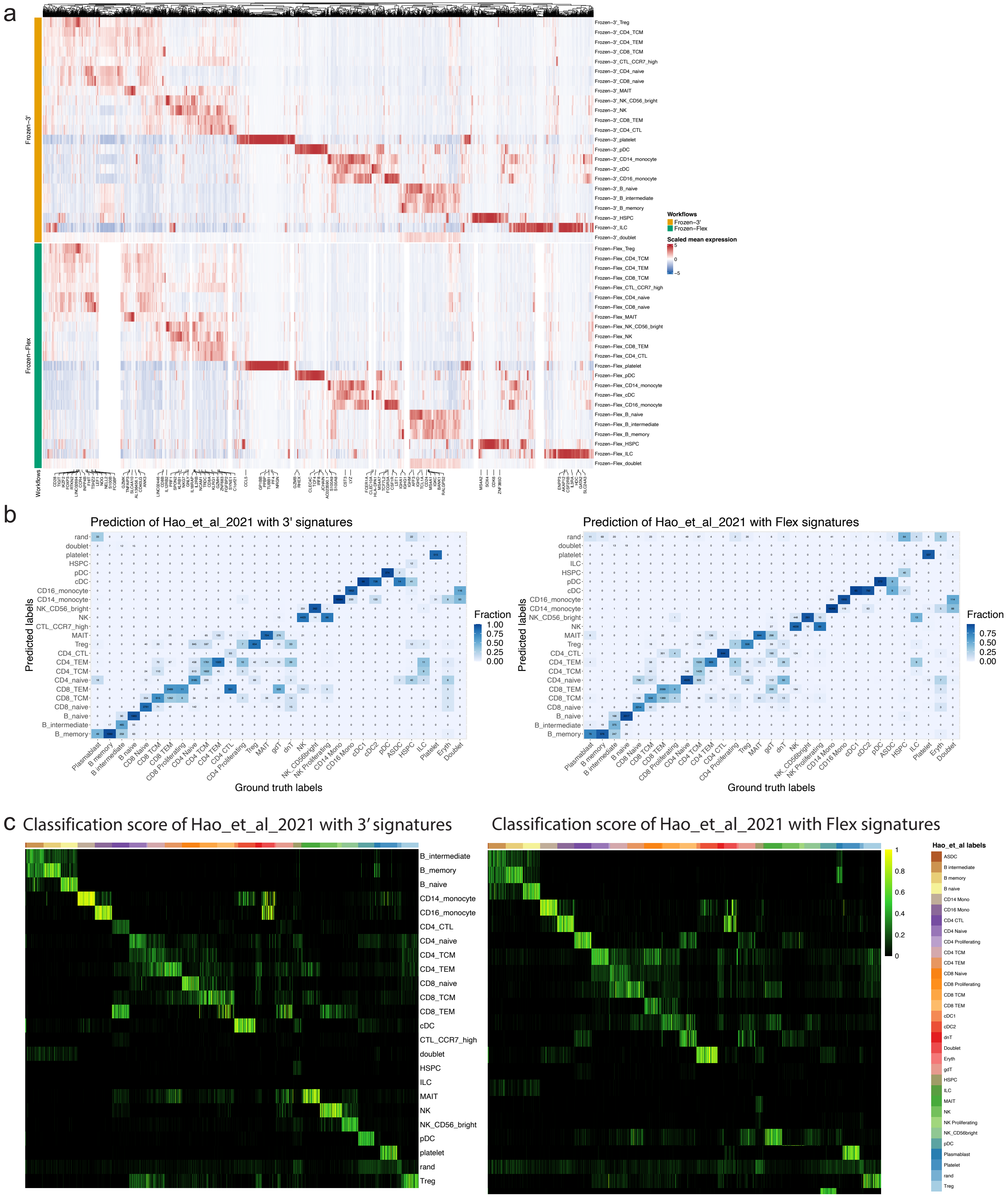
a
Frozen-3'
Frozen-Flex
Workflows
Frozen-3'
Frozen-Flex
Scaled mean expression
5
0
-5
Frozen-3'_Treg
Frozen-3'_CD4_TCM
Frozen-3'_CD4_TEM
Frozen-3'_CD8_TCM
Frozen-3'_CTL_CCR7_high
Frozen-3'_CD4_naive
Frozen-3'_CD8_naive
Frozen-3'_MAIT
Frozen-3'_NK_CD56_bright
Frozen-3'_NK
Frozen-3'_CD8_TEM
Frozen-3'_CD4_CTL
Frozen-3'_platelet
Frozen-3'_pDC
Frozen-3'_CD14_monocyte
Frozen-3'_cDC
Frozen-3'_CD16_monocyte
Frozen-3'_B_naive
Frozen-3'_B_intermediate
Frozen-3'_B_memory
Frozen-3'_HSPC
Frozen-3'_ILC
Frozen-3'_doublet
Frozen-Flex_Treg
Frozen-Flex_CD4_TCM
Frozen-Flex_CD4_TEM
Frozen-Flex_CD8_TCM
Frozen-Flex_CTL_CCR7_high
Frozen-Flex_CD4_naive
Frozen-Flex_CD8_naive
Frozen-Flex_MAIT
Frozen-Flex_NK_CD56_bright
Frozen-Flex_NK
Frozen-Flex_CD8_TEM
Frozen-Flex_CD4_CTL
Frozen-Flex_platelet
Frozen-Flex_pDC
Frozen-Flex_CD14_monocyte
Frozen-Flex_cDC
Frozen-Flex_CD16_monocyte
Frozen-Flex_B_naive
Frozen-Flex_B_intermediate
Frozen-Flex_B_memory
Frozen-Flex_HSPC
Frozen-Flex_ILC
Frozen-Flex_doublet
b
Prediction of Hao_et_al_2021 with 3' signatures
Prediction of Hao_et_al_2021 with Flex signatures
Predicted labels
Ground truth labels
Fraction
1.00
0.75
0.50
0.25
0.00
rand
doublet
platelet
HSPC
ILC
pDC
cDC
CD16_monocyte
CD14_monocyte
NK_CD56_bright
NK
CTL_CCR7_high
MAIT
Treg
CD4_CTL
CD4_TEM
CD4_TCM
CD4_naive
CD8_TEM
CD8_TCM
CD8_naive
B_naive
B_intermediate
B_memory
Plasmablast
B memory
B intermediate
B naive
CD8 Naive
CD8 TCM
CD8 TEM
CD8 Proliferating
CD4 Naive
CD4 TCM
CD4 TEM
CD4 CTL
CD4 Proliferating
Treg
MAIT
gdT
dnT
NK
NK CD56bright
NK Proliferating
CD14 Mono
CD16 Mono
cDC1
cDC2
pDC
ASDC
HSPC
ILC
Platelet
Eryth
Doublet
c
Classification score of Hao_et_al_2021 with 3' signatures
Classification score of Hao_et_al_2021 with Flex signatures
B_intermediate
B_memory
B_naive
CD14_monocyte
CD16_monocyte
CD4_CTL
CD4_naive
CD4_TCM
CD4_TEM
CD8_naive
CD8_TCM
CD8_TEM
cDC
CTL_CCR7_high
doublet
HSPC
ILC
MAIT
NK
NK_CD56_bright
pDC
platelet
rand
Treg
1
0.8
0.6
0.4
0.2
0
Hao_et_al labels
ASDC
B intermediate
B memory
B naive
CD14 Mono
CD16 Mono
CD4 CTL
CD4 Naive
CD4 Proliferating
CD4 TCM
CD4 TEM
CD8 Naive
CD8 Proliferating
CD8 TCM
CD8 TEM
cDC1
cDC2
dnT
Doublet
Eryth
gdT
HSPC
ILC
MAIT
NK
NK Proliferating
NK_CD56bright
pDC
Plasmablast
Platelet
rand
Treg

Supplementary Figure 2: 10x Flex chemistry detects comparable cell type signatures as the 10x 3' chemistry. (**a**) Heatmap of the scaled expression of top differentially expressed genes detected between cell types in the integrated PBMC data. The top 200 significantly differentially expressed genes identified in each cell type (if available) were selected by fold change and used for plotting. A gene was considered significantly differentially expressed if the BH-adjusted P value was lower than 0.05. Genes were arranged by hierarchical clustering based on the expression in the FFPE-snPATHO-seq data on the x-axis. Cell types identified by different snRNA-seq workflows were manually arranged on the y-axis. Genes not targeted by the Flex probe panel were also included and assigned a fold change of 0 in the plot. (**b**) Heatmap showing the concordance between the published PBMC cell type annotations obtained from Hao et al. 2021 and the cell type annotations predicted using the gene expression signatures from the 3' or Flex data collected in this study. (**c**) Heatmap of the cell type classification confidence scores when the 3' or Flex data collected from this study was used to annotate the published PBMC data.

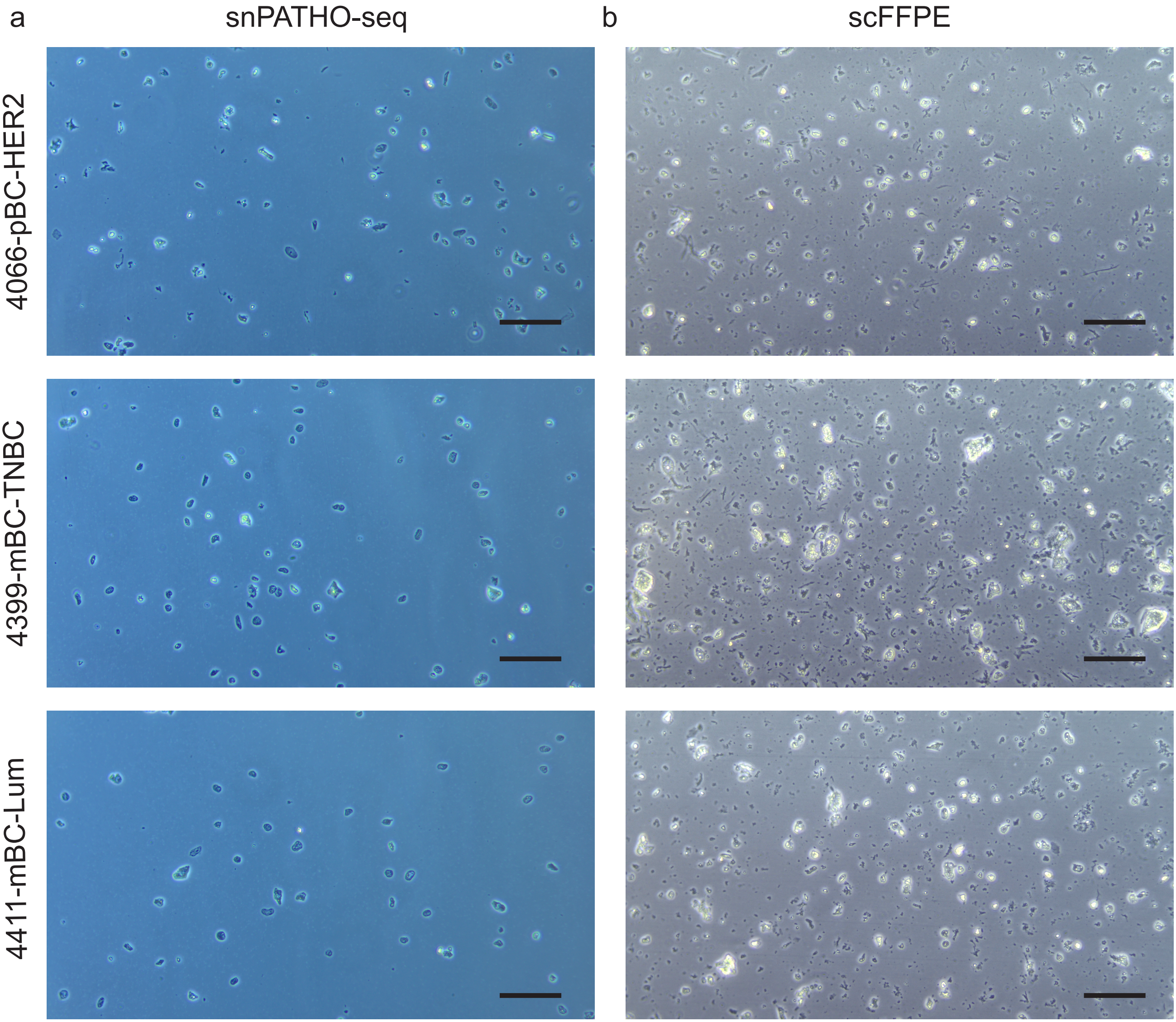

Supplementary Figure 3: Assessment of the quality of tissue dissociation products generated by the snPATHO-seq and the scFFPE workflows. (**a**) Representative phase-contrast microscopic images of the nuclei extracted using the snPATHO-seq workflow from the 4066, 4399, and 4411. (**b**) Representative phase-contrast microscopic images of the tissue dissociation products prepared using the scFFPE workflow from the 4066, 4399, and 4411. Scale bars = 100μm.

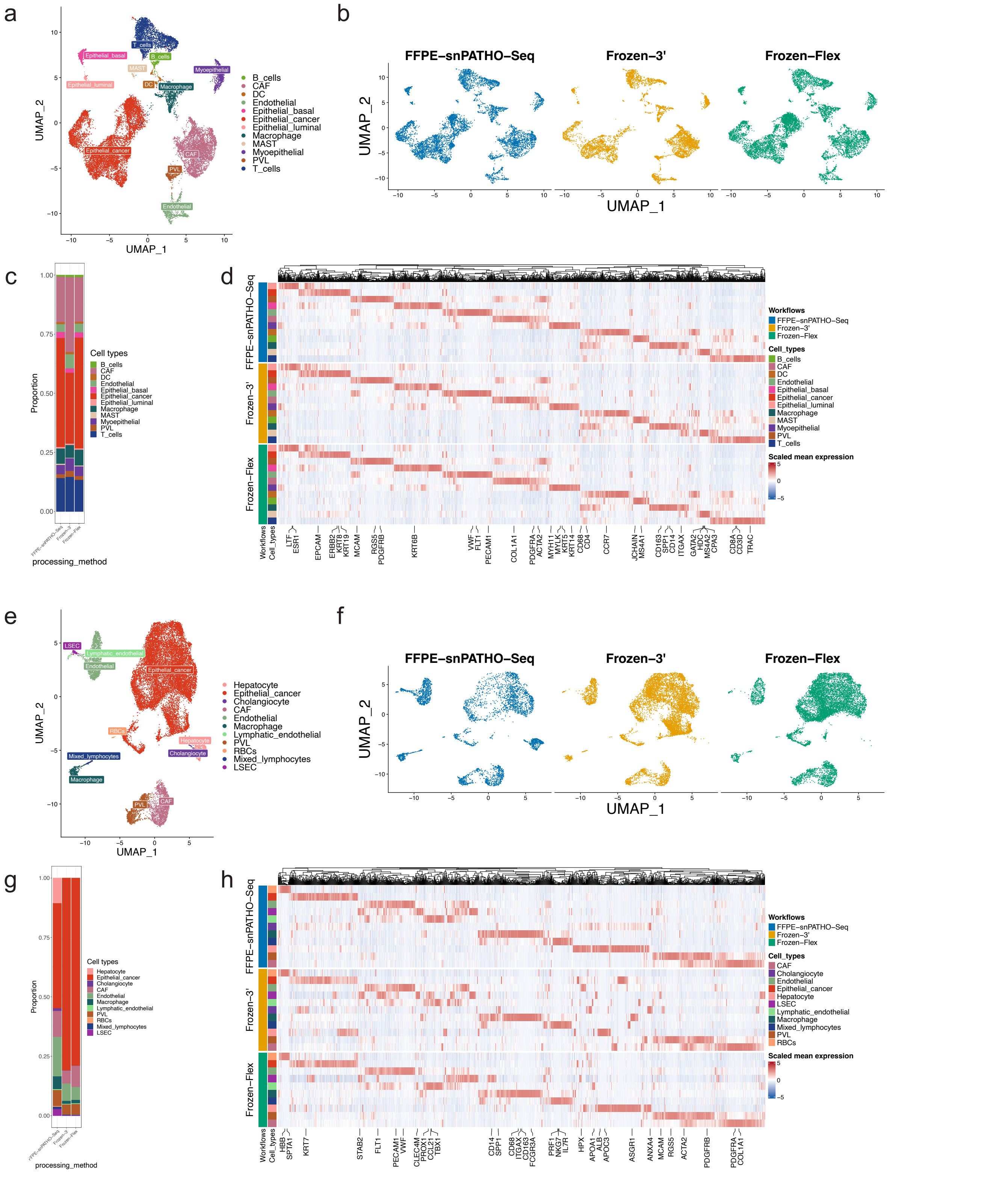
a
b
c
d
e
f
g
h
UMAP_1
UMAP_2
FFPE–snPATHO–Seq
Frozen–3'
Frozen–Flex
B_cells
CAF
DC
Endothelial
Epithelial_basal
Epithelial_cancer
Epithelial_luminal
Macrophage
MAST
Myoepithelial
PVL
T_cells
Cell types
Proportion
processing_method
Workflows
Cell_types
Scaled mean expression
LTF
ESR1
EPCAM
ERBB2
KRT8
KRT19
MCAM
RGS5
PDGFRB
KRT6B
VWF
FLT1
PECAM1
COL1A1
PDGFRA
ACTA2
MYH11
MYLK
KRT5
KRT14
CD68
CD4
CCR7
JCHAIN
MS4A1
CD163
SPP1
CD14
ITGAX
GATA2
HDC
MS4A2
CPA3
CD8A
CD3D
TRAC
Hepatocyte
Cholangiocyte
Lymphatic_endothelial
RBCs
Mixed_lymphocytes
LSEC
HBB
SPTA1
KRT7
STAB2
CLEC4M
PROX1
CCL21
TBX1
FCGR3A
PRF1
NKG7
IL7R
HPX
APOA1
ALB
APOC3
ASGR1
ANXA4

Supplementary Figure 4: snRNA-seq methods detected overall comparable transcriptomic features and cell type representations in samples 4066 and 4399. (**a,e**) UMAP embedding of Seurat CCA integrated snRNA-seq data with cell type annotation from 4066 (**a**) and 4399 (**e**). (**b,f**) UMAP embedding of Seruat CCA integrated snRNA-seq data split by processing methods from 4066 (**b**) and 4399 (**f**). (**c,g**) Barplots of the fraction of cell types detected by different snRNA-seq methods from 4066 (**c**) and 4399 (**g**). (**d,h**) Heatmaps of the scaled expression of top differentially expressed genes between cell types from 4066 (**d**) and 4399 (**h**). The top 200 significantly differentially expressed genes identified in each cell type (if available) were selected by fold change and used for plotting. A gene was considered significantly differentially expressed if the BH-adjusted P value was lower than 0.05. Genes were arranged by hierarchical clustering by expression in the FFPE-snPATHO-seq data on the x-axis. Cell types identified by different snRNA-seq workflows were manually arranged on the y-axis.

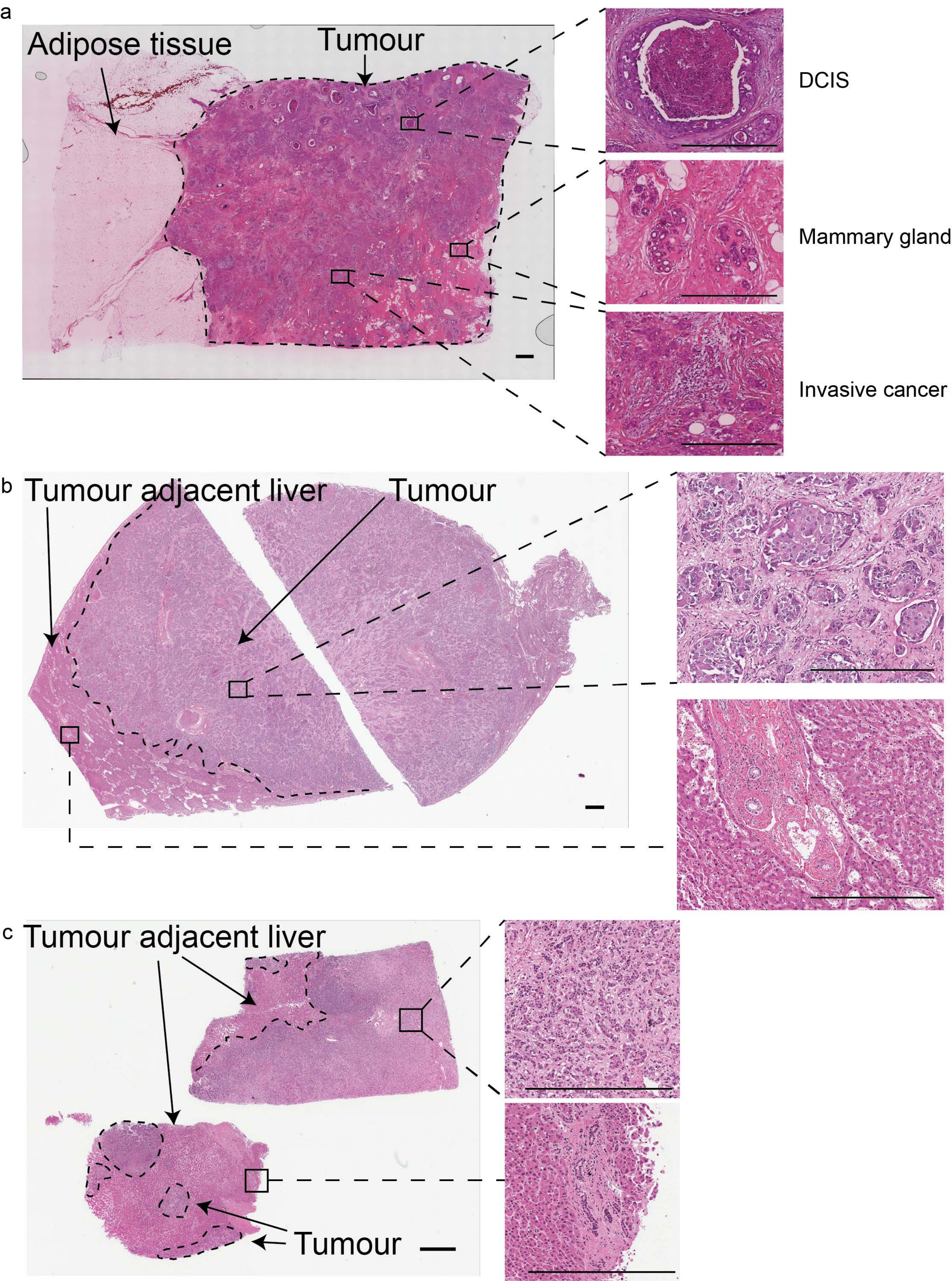

a
Adipose tissue
Tumour
DCIS
Mammary gland
Invasive cancer
b
Tumour adjacent liver
Tumour
c
Tumour adjacent liver
Tumour

Supplementary Figure 5: Histopathological features of the breast cancer FFPE samples tested. (**a**) H&E images of the whole tissue scan and high-magnification images of selected DCIS, normal mammary gland, and invasive cancer regions for sample 4066. (**b**) H&E images of the whole tissue scan and high-magnification images of selected invasive cancer and normal liver regions for sample 4399. (**c**) H&E images of the whole tissue scan and high-magnification images of selected invasive cancer and normal liver regions for sample 4411. Scale bars = 1mm.

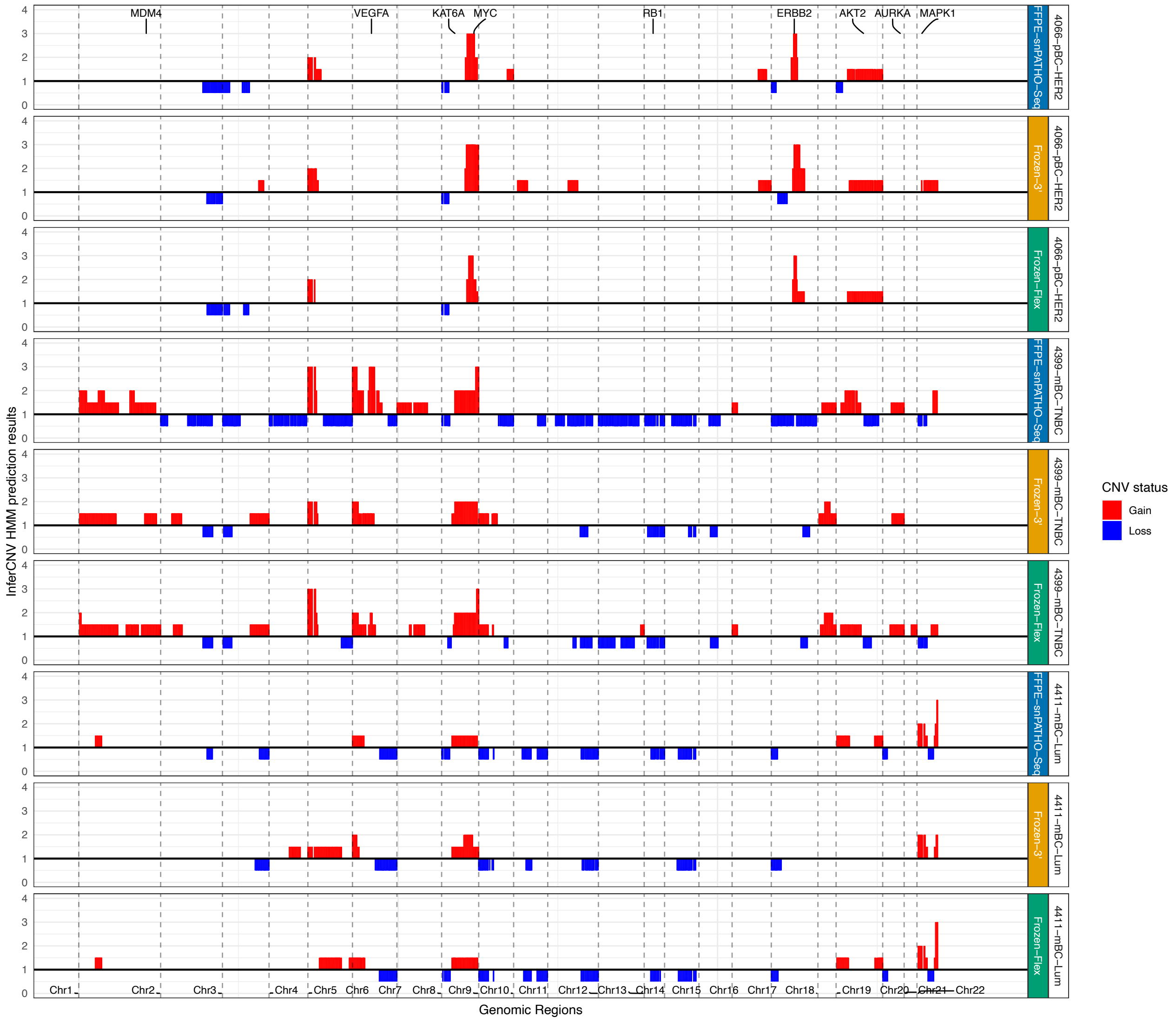

Supplementary Figure 6: the snPATHO-seq workflow detected comparable cancer CNV patterns as the other snRNA-seq methods tested. Line plot of the summarised CNV patterns inferred using each dataset. Only one CNV pattern was detected among the cancer cells in each snRNA-seq dataset (per patient per snRNA-seq method). The average CNV profiles of all cancer nuclei in each dataset were calculated and used for visualization.

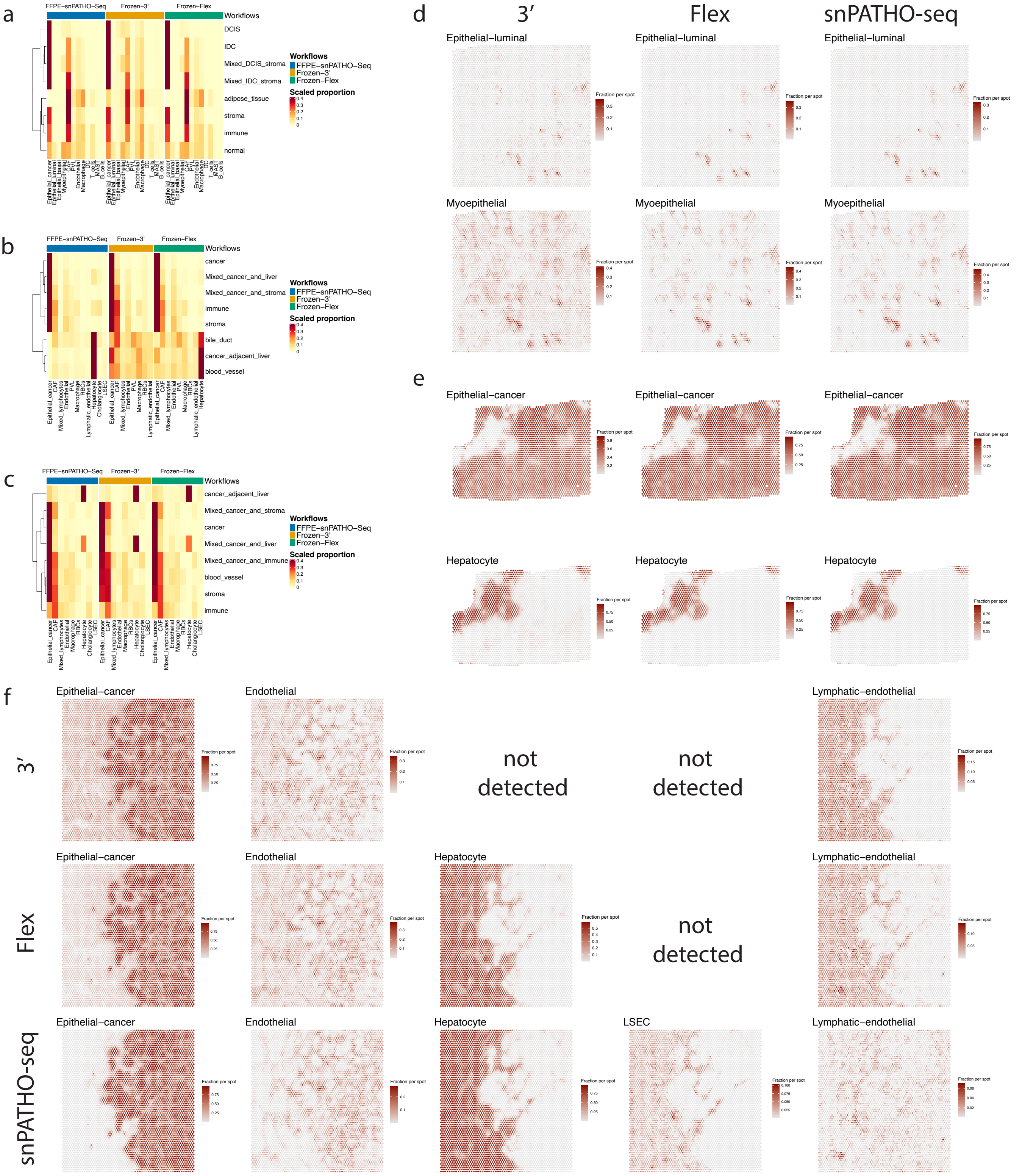
a
FFPE–snPATHO–Seq
Frozen–3'
Frozen–Flex
Workflows
DCIS
IDC
Mixed_DCIS_stroma
Mixed_IDC_stroma
adipose_tissue
stroma
immune
normal
Epithelial_cancer
Epithelial_luminal
Epithelial_basal
Myoepithelial
CAF
PVL
Endothelial
Macrophage
DC
T_cells
MAST
B_cells
Workflows
FFPE–snPATHO–Seq
Frozen–3'
Frozen–Flex
Scaled proportion
0.4
0.3
0.2
0.1
0
b
cancer
Mixed_cancer_and_liver
Mixed_cancer_and_stroma
immune
stroma
bile_duct
cancer_adjacent_liver
blood_vessel
Epithelial_cancer
CAF
Mixed_lymphocytes
Endothelial
PVL
Macrophage
RBCs
Lymphatic_endothelial
Hepatocyte
Cholangiocyte
LSEC
c
cancer_adjacent_liver
Mixed_cancer_and_stroma
cancer
Mixed_cancer_and_liver
Mixed_cancer_and_immune
blood_vessel
stroma
immune
d
3′
Flex
snPATHO-seq
Epithelial–luminal
Myoepithelial
Fraction per spot
e
Epithelial–cancer
Hepatocyte
f
3′
Flex
snPATHO-seq
Epithelial–cancer
Endothelial
Hepatocyte
LSEC
Lymphatic–endothelial
not detected

Supplementary Figure 7: snPATHO-seq allows accurate inference of spatial cellular composition of matching FFPE Visium data. (**a–c**) Heatmaps of the fraction of snRNA-seq cell types inferred across different spatial domains for samples 4066 (**a**), 4399 (**b**), and 4411 (**c**). Spatial domains were annotated by a pathologist using the H&E image. (**d**) Spatial distributions of the luminal epithelial and myoepithelial cell signatures inferred using the data generated with different snRNA-seq methods on the same FFPE Visium data for patient 4066. (**e**) Spatial distributions of the epithelial cancer and hepatocyte signatures inferred using the data generated with different snRNA-seq methods on the same FFPE Visium data for patient 4411. (**f**) Spatial distributions of the epithelial cancer, hepatocyte, and endothelial cell signatures inferred using the data generated with different snRNA-seq methods on the same FFPE Visium data for patient 4399.

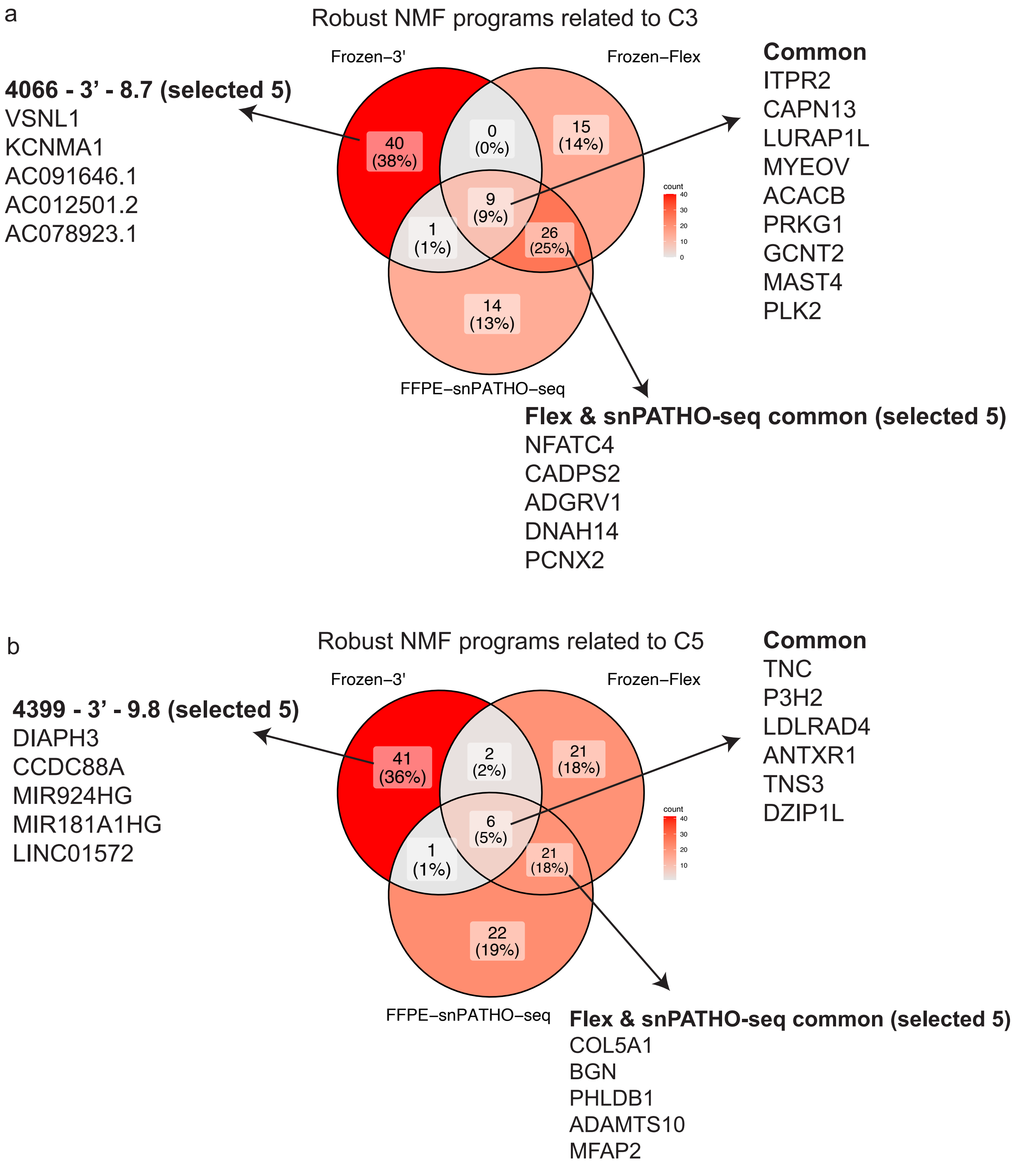
a
Robust NMF programs related to C3
Frozen−3'
Frozen−Flex
FFPE−snPATHO−seq
40
(38%)
0
(0%)
15
(14%)
9
(9%)
1
(1%)
26
(25%)
14
(13%)
count
40
30
20
10
0
4066 - 3’ - 8.7 (selected 5)
VSNL1
KCNMA1
AC091646.1
AC012501.2
AC078923.1
Common
ITPR2
CAPN13
LURAP1L
MYEOV
ACACB
PRKG1
GCNT2
MAST4
PLK2
Flex & snPATHO-seq common (selected 5)
NFATC4
CADPS2
ADGRV1
DNAH14
PCNX2
b
Robust NMF programs related to C5
Frozen−3'
Frozen−Flex
FFPE−snPATHO−seq
41
(36%)
2
(2%)
21
(18%)
6
(5%)
1
(1%)
21
(18%)
22
(19%)
count
40
30
20
10
4399 - 3’ - 9.8 (selected 5)
DIAPH3
CCDC88A
MIR924HG
MIR181A1HG
LINC01572
Common
TNC
P3H2
LDLRAD4
ANTXR1
TNS3
DZIP1L
Flex & snPATHO-seq common (selected 5)
COL5A1
BGN
PHLDB1
ADAMTS10
MFAP2

Supplementary Figure 8: the snPATHO-seq and 10x Flex workflows detected more comparable cancer transcriptomic signatures than the 10x 3' workflow. (**a**) Venn diagram of the number of overlapping genes between robust NMF programs related to the calcium signature (C3) derived using different snRNA-seq methods. Common genes shared across all robust NMF programs, those (selected 5) only included in the robust NMF programs from the 3' workflow, and those (selected 5) shared between the robust NMF programs from the Flex and the snPATHO-seq data were highlighted. (**b**) Venn diagram of the number of overlapping genes between robust NMF programs related to the ECM organization (C5) signature derived using different snRNA-seq methods. Common genes shared across all robust NMF programs, those (selected 5) only included in the robust NMF programs from the 3' workflow, and those (selected 5) shared between the robust NMF programs from the Flex and the snPATHO-seq data were highlighted.

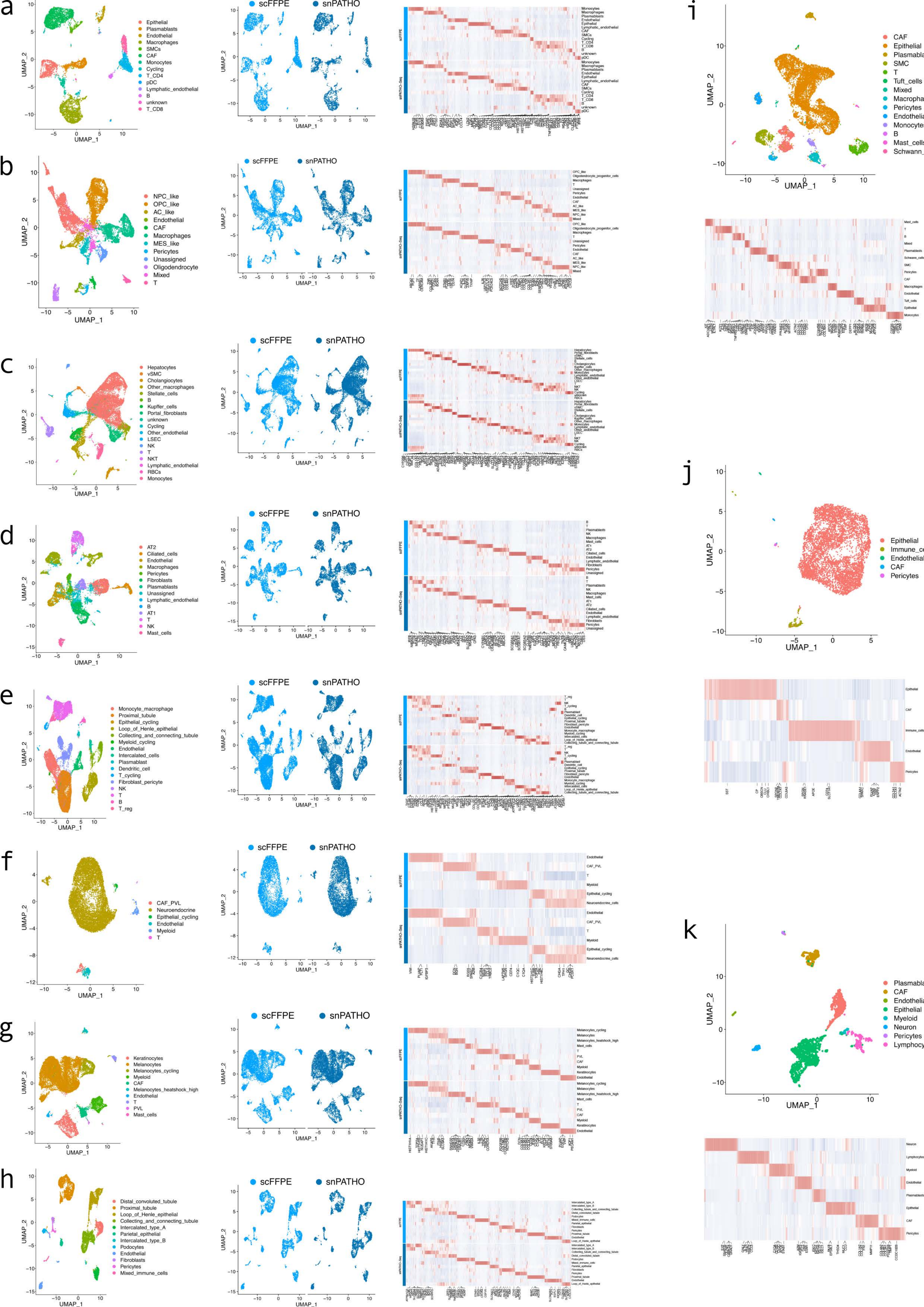
a
Epithelial
Plasmablasts
Endothelial
Macrophages
SMCs
CAF
Monocytes
Cycling
T_CD4
pDC
Lymphatic_endothelial
B
unknown
T_CD8
UMAP_1
UMAP_2
scFFPE
snPATHO
snPATHO-Seq
b
NPC_like
OPC_like
AC_like
Endothelial
CAF
Macrophages
MES_like
Pericytes
Unassigned
Oligodendrocyte
Mixed
T
c
Hepatocytes
vSMC
Cholangiocytes
Other_macrophages
Stellate_cells
B
Kupffer_cells
Portal_fibroblasts
unknown
Cycling
Other_endothelial
LSEC
NK
T
NKT
Lymphatic_endothelial
RBCs
Monocytes
d
AT2
Ciliated_cells
Endothelial
Macrophages
Pericytes
Fibroblasts
Plasmablasts
Unassigned
Lymphatic_endothelial
B
AT1
T
NK
Mast_cells
e
Monocyte_macrophage
Proximal_tubule
Epithelial_cycling
Loop_of_Henle_epithelial
Collecting_and_connecting_tubule
Myeloid_cycling
Endothelial
Intercalated_cells
Plasmablast
Dendritic_cell
T_cycling
Fibroblast_pericyte
NK
T
B
T_reg
f
CAF_PVL
Neuroendocrine
Epithelial_cycling
Endothelial
Myeloid
T
g
Keratinocytes
Melanocytes
Melanocytes_cycling
Myeloid
CAF
Melanocytes_heatshock_high
Endothelial
T
PVL
Mast_cells
h
Distal_convoluted_tubule
Proximal_tubule
Loop_of_Henle_epithelial
Collecting_and_connecting_tubule
Intercalated_type_A
Parietal_epithelial
Intercalated_type_B
Podocytes
Endothelial
Fibroblasts
Pericytes
Mixed_immune_cells
i
CAF
Epithelial
Plasmabla
SMC
T
Tuft_cells
Mixed
Macropha
Pericytes
Endothelia
Monocyte
B
Mast_cells
Schwann_
j
Epithelial
Immune_c
Endothelia
CAF
Pericytes
k
Plasmabla
CAF
Endothelia
Epithelial
Myeloid
Neuron
Pericytes
Lymphocy

Supplementary Figure 9: Overview of snPATHO-seq and scFFPE data collected from FFPE tissue samples other than breast cancer. (**a–h**) Cell type annotation, data integration, and the expression of top differentially expressed genes of Endocervical_Adenocarcinoma_6707 (**a**), Glioblastoma_1773A (**b**), liver_8754A (**c**), lung_20011329NL (**d**), Kidney_K48 (**e**), lung_cancer_20011329LC (**f**), melanoma_7167A (**g**), and Kidney_1305272B (**h**). (**i–k**) Cell type annotation and the expression of top differentially expressed markers of Colon_213641(**i**), Endometrium_220952 (**j**), and Ovary_230303 (**k**). For the construction of heatmaps, the top 200 significantly differentially expressed genes identified in each cell type (if available) were selected by fold change and used for plotting. A gene was considered significantly differentially expressed if the BH-adjusted P value was lower than 0.05. Genes were arranged by hierarchical clustering by expression in the FFPE-snPATHO-seq data on the x-axis. Cell types identified by different snRNA-seq workflows were manually arranged on the y-axis.

Supplementary table 1: correspondance of robust NMF programs to clusters

plot_order	Cluster on the heatmap
4066_FFPE_Epithelial_cancer_rank4_9_nruns10.RDS.8.8	C1
4066_FLEX_Epithelial_cancer_rank4_9_nruns10.RDS.9.5	C1
4066_3p_Epithelial_cancer_rank4_9_nruns10.RDS.9.2	
4411_3p_Epithelial_cancer_rank4_9_nruns10.RDS.7.6	C2
4411_FLEX_Epithelial_cancer_rank4_9_nruns10.RDS.7.5	C2
4411_FFPE_Epithelial_cancer_rank4_9_nruns10.RDS.6.2	C2
4066_3p_Epithelial_cancer_rank4_9_nruns10.RDS.8.7	
4066_FFPE_Epithelial_cancer_rank4_9_nruns10.RDS.6.1	C3
4066_FLEX_Epithelial_cancer_rank4_9_nruns10.RDS.6.1	C3
4411_3p_Epithelial_cancer_rank4_9_nruns10.RDS.7.5	C4
4411_FFPE_Epithelial_cancer_rank4_9_nruns10.RDS.7.6	C4
4411_FLEX_Epithelial_cancer_rank4_9_nruns10.RDS.7.2	C4
4399_3p_Epithelial_cancer_rank4_9_nruns10.RDS.9.8	
4399_FLEX_Epithelial_cancer_rank4_9_nruns10.RDS.7.2	C5
4399_FFPE_Epithelial_cancer_rank4_9_nruns10.RDS.9.5	C5
4411_3p_Epithelial_cancer_rank4_9_nruns10.RDS.5.2	C6
4066_3p_Epithelial_cancer_rank4_9_nruns10.RDS.4.2	C6
4399_FLEX_Epithelial_cancer_rank4_9_nruns10.RDS.6.6	C6
4399_FFPE_Epithelial_cancer_rank4_9_nruns10.RDS.4.3	C6
4066_FLEX_Epithelial_cancer_rank4_9_nruns10.RDS.5.3	C6
4066_FFPE_Epithelial_cancer_rank4_9_nruns10.RDS.4.1	C6
4411_FFPE_Epithelial_cancer_rank4_9_nruns10.RDS.5.3	C6
4411_FLEX_Epithelial_cancer_rank4_9_nruns10.RDS.5.5	C6
4411_FLEX_Epithelial_cancer_rank4_9_nruns10.RDS.6.4	
4411_FFPE_Epithelial_cancer_rank4_9_nruns10.RDS.7.7	
4399_3p_Epithelial_cancer_rank4_9_nruns10.RDS.7.2	C7
4399_FFPE_Epithelial_cancer_rank4_9_nruns10.RDS.7.5	C7
4399_FLEX_Epithelial_cancer_rank4_9_nruns10.RDS.8.8	C7

4411_3p_Epithelial_cancer_rank4_9_nruns10.RDS.6.2	C8
4411_FLEX_Epithelial_cancer_rank4_9_nruns10.RDS.7.3	C8
4411_FFPE_Epithelial_cancer_rank4_9_nruns10.RDS.7.3	C8
4066_3p_Epithelial_cancer_rank4_9_nruns10.RDS.6.6	C9
4066_FFPE_Epithelial_cancer_rank4_9_nruns10.RDS.7.5	C9
4066_FLEX_Epithelial_cancer_rank4_9_nruns10.RDS.7.2	C9
4399_3p_Epithelial_cancer_rank4_9_nruns10.RDS.9.3	C10
4411_3p_Epithelial_cancer_rank4_9_nruns10.RDS.6.1	C10
4399_FFPE_Epithelial_cancer_rank4_9_nruns10.RDS.6.4	C10
4399_FLEX_Epithelial_cancer_rank4_9_nruns10.RDS.9.3	C10
4411_FFPE_Epithelial_cancer_rank4_9_nruns10.RDS.7.4	C10
4411_FLEX_Epithelial_cancer_rank4_9_nruns10.RDS.7.7	C10
4399_3p_Epithelial_cancer_rank4_9_nruns10.RDS.7.6	
4066_3p_Epithelial_cancer_rank4_9_nruns10.RDS.8.8	C11
4399_FFPE_Epithelial_cancer_rank4_9_nruns10.RDS.5.1	C11
4411_FLEX_Epithelial_cancer_rank4_9_nruns10.RDS.8.6	C11
4411_FFPE_Epithelial_cancer_rank4_9_nruns10.RDS.9.1	C11
4066_FFPE_Epithelial_cancer_rank4_9_nruns10.RDS.9.1	C11
4066_FLEX_Epithelial_cancer_rank4_9_nruns10.RDS.8.1	C11
4066_FLEX_Epithelial_cancer_rank4_9_nruns10.RDS.8.8	C12
4066_FFPE_Epithelial_cancer_rank4_9_nruns10.RDS.9.2	C12
4411_FLEX_Epithelial_cancer_rank4_9_nruns10.RDS.7.1	C13
4411_FFPE_Epithelial_cancer_rank4_9_nruns10.RDS.8.8	C13
4399_3p_Epithelial_cancer_rank4_9_nruns10.RDS.4.3	C14
4411_3p_Epithelial_cancer_rank4_9_nruns10.RDS.8.2	C14
4066_FLEX_Epithelial_cancer_rank4_9_nruns10.RDS.5.5	C15
4066_FFPE_Epithelial_cancer_rank4_9_nruns10.RDS.4.4	C15
4399_FFPE_Epithelial_cancer_rank4_9_nruns10.RDS.4.4	C15
4399_FLEX_Epithelial_cancer_rank4_9_nruns10.RDS.4.3	C15
