# Supplementary material for: snPATHO-seq, a versatile FFPE single-nucleus RNA sequencing method to unlock pathology archives
Source: Commun Biol. 2024 Oct 16;7:1340. doi: 10.1038/s42003-024-07043-2 (PMC11484811; doi:10.1038/s42003-024-07043-2)
Supplement: Supplementary file 3 — Description of Additional Supplementary Materials [file 42003_2024_7043_MOESM3_ESM.pdf]

Description of Additional Supplementary Files

File name: Supplementary Data 1

Description: Clinical meta data of all samples processed

File name: Supplementary Data 2

Description: robust NMF gene modules derived from snRNA-seq and Visium data

File name: Supplementary Data 3

Description: cores genes of each robust NMF clusters (core genes: genes present in at least 2 robust NMF programs within each cluster)
