# Supplementary material for: snPATHO-seq, a versatile FFPE single-nucleus RNA sequencing method to unlock pathology archives
Source: Commun Biol. 2024 Oct 16;7:1340. doi: 10.1038/s42003-024-07043-2 (PMC11484811; doi:10.1038/s42003-024-07043-2)
Supplement: Supplementary file 7 — Reporting Summary [file 42003_2024_7043_MOESM7_ESM.pdf]

nature portfolio

Corresponding author(s): Luciano Martelotto, Alexander Swarbrick

Last updated by author(s): 2024/09/30

Reporting Summary

Nature Portfolio wishes to improve the reproducibility of the work that we publish. This form provides structure for consistency and transparency in reporting. For further information on Nature Portfolio policies, see our Editorial Policies and the Editorial Policy Checklist.

Statistics

For all statistical analyses, confirm that the following items are present in the figure legend, table legend, main text, or Methods section.

n/a	Confirmed	
☐	☒	The exact sample size (*n*) for each experimental group/condition, given as a discrete number and unit of measurement
☐	☒	A statement on whether measurements were taken from distinct samples or whether the same sample was measured repeatedly
☐	☒	The statistical test(s) used AND whether they are one- or two-sided<br>*Only common tests should be described solely by name; describe more complex techniques in the Methods section.*
☐	☒	A description of all covariates tested
☐	☒	A description of any assumptions or corrections, such as tests of normality and adjustment for multiple comparisons
☐	☒	A full description of the statistical parameters including central tendency (e.g. means) or other basic estimates (e.g. regression coefficient) AND variation (e.g. standard deviation) or associated estimates of uncertainty (e.g. confidence intervals)
☐	☒	For null hypothesis testing, the test statistic (e.g. *F*, *t*, *r*) with confidence intervals, effect sizes, degrees of freedom and *P* value noted<br>*Give P values as exact values whenever suitable.*
☒	☐	For Bayesian analysis, information on the choice of priors and Markov chain Monte Carlo settings
☒	☐	For hierarchical and complex designs, identification of the appropriate level for tests and full reporting of outcomes
☒	☐	Estimates of effect sizes (e.g. Cohen's *d*, Pearson's *r*), indicating how they were calculated

Our web collection on statistics for biologists contains articles on many of the points above.

Software and code

Policy information about availability of computer code

Data collection	No specific software was used for the collection of the data.
Data analysis	scRNA-seq / snRNA-seq FASTQ file alignment was performed using Cellranger v2023.0415.0 / Cellranger v7.0.1, GRCh38 build 2020-A, 10X Genomics and Chromium Human Transcriptome Probe Set v1.0.1. Additional data analysis was performed using R v4.1.1, Seurat v4.3.0.9002, DoubletFinder v2.0.3, singleCellNet v0.1.0, inferCNV v1.10.1, NMF v0.26 (with R 4.2.3) and fgsea v1.20.0.<br><br>Spatial transcriptomics data alignment was performed using Spaceranger v2.0.0, GRCh38 (build 2020-A, 10X Genomics) and Visium Human Transcriptome Probe Set v2.0. Pathology annotation was performed using Loupe browser (v6.2.0, 10X Genomics). Additional data analysis was performed using R 4.1.1, STutility v1.1.1 and spacexr v2.2.0.

For manuscripts utilizing custom algorithms or software that are central to the research but not yet described in published literature, software must be made available to editors and reviewers. We strongly encourage code deposition in a community repository (e.g. GitHub). See the Nature Portfolio guidelines for submitting code & software for further information.

Data

Policy information about availability of data

All manuscripts must include a data availability statement. This statement should provide the following information, where applicable:

- Accession codes, unique identifiers, or web links for publicly available datasets
- A description of any restrictions on data availability
- For clinical datasets or third party data, please ensure that the statement adheres to our policy

A data availability statement has been included in the manuscript. The accession code to data has been provided. Public access to the source data has been granted.

Research involving human participants, their data, or biological material

Policy information about studies with human participants or human data. See also policy information about sex, gender (identity/presentation), and sexual orientation and race, ethnicity and racism.

Reporting on sex and gender	Sex and gender information was not collected or used for experiment design or data analysis.
Reporting on race, ethnicity, or other socially relevant groupings	Socially grouping information was not collected or used for experiment design or data analysis.
Population characteristics	Population characteristics information was not collected or used for experiment design or data analysis.
Recruitment	This study is based on archival tissue sample and no new patient was recruited.<br><br>For breast cancer patients, patients who have matching FFPE and snap frozen samples from the same lesion preserved were selected to support the comparison made in this study.
Ethics oversight	St Vincent's Hospital Ethics Committee (protocol SVH 17/173)<br>St Vincent's Hospital Ethics Committee (protocol X19-0496)<br>Ethical review board of Centre Léon Bérard (protocols - endometrium: 2022-05, colon: 2021-33, ovary: I-3422-01_MTA_IP_CLB)<br>Other human samples were sourced from commercial source as highlighted in the supplementary table S1.

Note that full information on the approval of the study protocol must also be provided in the manuscript.

Field-specific reporting

Please select the one below that is the best fit for your research. If you are not sure, read the appropriate sections before making your selection.

☒ Life sciences ☐ Behavioural & social sciences ☐ Ecological, evolutionary & environmental sciences

For a reference copy of the document with all sections, see nature.com/documents/nr-reporting-summary-flat.pdf

Life sciences study design

All studies must disclose on these points even when the disclosure is negative.

Sample size	Sample size was determined by the availability of human samples and research funding.
Data exclusions	For the PBMC / breast cancer datasets, the scRNA-seq (PBMC) / snRNA-seq (breast cancer) data was downsampled to around 30,000 / 25,000 reads per cell for the direct comparison of performance.<br><br>For all snRNA-seq data, cellranger outputs were filtered with the cellbender v.0.2.0 to exclude ambient counts in the gene expression matrix.<br><br>Any nuclei with < 200 genes, > 8000 genes or > 10% mitochondrial gene product were defined as low quality nuclei and excluded.<br><br>Additional doublet filtering was performed using the DoubletFinder package following default settings.<br><br>Detailed description of data filtering and processing can be found in the method section of the manuscript.
Replication	The datasets generated in this study represented a single independent replicate per patient tumour. This was primarily due to constrains in sample and funding availability.
Randomization	Randomization was not performed.
Blinding	Blinding was not performed.

Reporting for specific materials, systems and methods

We require information from authors about some types of materials, experimental systems and methods used in many studies. Here, indicate whether each material, system or method listed is relevant to your study. If you are not sure if a list item applies to your research, read the appropriate section before selecting a response.

Materials & experimental systems

n/a	Involved in the study
☒	☐ Antibodies
☒	☐ Eukaryotic cell lines
☒	☐ Palaeontology and archaeology
☒	☐ Animals and other organisms
☒	☐ Clinical data
☒	☐ Dual use research of concern
☒	☐ Plants

Methods

n/a	Involved in the study
☒	☐ ChIP-seq
☒	☐ Flow cytometry
☒	☐ MRI-based neuroimaging

Plants

Seed stocks	n/a
Novel plant genotypes	n/a
Authentication	n/a
